# miRNA Expression Patterns in Early- and Late-Stage Prostate Cancer Patients: High-Throughput Analysis

**DOI:** 10.3390/biomedicines11113073

**Published:** 2023-11-16

**Authors:** Irina Gilyazova, Elizaveta Ivanova, Himanshu Gupta, Artur Mustafin, Ruslan Ishemgulov, Adel Izmailov, Gulshat Gilyazova, Elena Pudova, Valentin Pavlov, Elza Khusnutdinova

**Affiliations:** 1 Subdivision of the Ufa Federal Research Centre of the Russian Academy of Sciences, Institute of Biochemistry and Genetics, 450054 Ufa, Russia; lizavetaivanova91@gmail.com (E.I.);; 2 Institute of Urology and Clinical Oncology, Department of Medical Genetics and Fundamental Medicine, Bashkir State Medical University, 450008 Ufa, Russia; 3 Biology Department, St. Petersburg State University, 199034 Saint-Petersburg, Russia; 4 Department of Biotechnology, Institute of Applied Sciences and Humanities, GLA University, Mathura 281406, India; himanshu.gupta@gla.ac.in; 5 Engelhardt Institute of Molecular Biology, Russian Academy of Sciences, 119991 Moscow, Russia

**Keywords:** prostate cancer, microRNA, gene expression

## Abstract

Prostate cancer (PCa) is one of the most common types of cancer among men. To date, there have been no specific markers identified for the diagnosis and prognosis or response to treatment of this disease. Thus, there is an urgent need for promising markers, which may be fulfilled by small non-coding RNAs known as microRNAs (miRNAs). Therefore, the present study aimed to investigate the miRNA profile in tissue samples obtained from patients with PCa using microarrays, followed by reverse transcriptase quantitative PCRs (RT-qPCRs). In the discovery phase, 754 miRNAs were screened in tissues obtained from patients (*n* = 46) with PCa in early and late stages. Expression levels of miRNA-324-3p, miRNA-429, miRNA-570, and miRNA-616 were found to be downregulated, and miRNA-423-5p expression was upregulated in patients with early-stage cancer compared to the late-stage ones. These five miRNAs were further validated in an independent cohort of samples (*n* = 39) collected from patients with PCa using RT-qPCR-based assays. MiRNA-324-3p, miRNA-429, miRNA-570, and miRNA-616 expression levels remained significantly downregulated in early-stage cancer tissues compared to late-stage tissues. Remarkably, for a combination of three miRNAs, PSA levels and Gleason scores were able to discriminate between patients with early-stage PCa and late-stage PCa, with an AUC of 95%, a sensitivity of 86%, and a specificity close to 94%. Thus, the data obtained in this study suggest a possible involvement of the identified miRNAs in the pathogenesis of PCa, and they may also have the potential to be developed into diagnostic and prognostic tools for PCa. However, further studies with a larger cohort are needed.

## 1. Introduction

Prostate cancer (PCa) is the second most common cancer in men, with an ever-increasing incidence. Despite its high morbidity, PCa is not highly lethal, and in most patients, it progresses slowly (indolent). There are several methods for PCa diagnostics and screening, including the prostate-specific antigen blood test (PSA). While this test reduces PCa mortality, it is associated with overdiagnosis, leading to unnecessary biopsies and treatment. Other diagnostic methods include digital rectal examination, trans-rectal ultrasound, and magnetic resonance imaging (MRI) fusion biopsy [1]. Unfortunately, often even with a biopsy, there is no absolute certainty that PCa will be detected. Furthermore, despite a fairly wide range of available methods, no approaches or markers have been developed to reliably distinguish indolent forms of PCa from aggressive ones [2]. Consequently, the screening for PCa stands as one of the most contentious subjects in the urologic literature, and it is presently not endorsed in most countries globally [3]. Numerous attempts are being made to develop other methods that could identify not only patients with PCa in general but also those with clinically significant disease. These methods aim to distinguish aggressive PCa from non-aggressive cases with high sensitivity and specificity. This approach would increase the coverage for those patients who need treatment immediately [4]. It should be recognized that, to date, this problem remains unsolved, and personalized approaches to managing PCa patients have not yet been developed. One marker used to distinguish a patient with PCa from a healthy individual is the urine PCA3 test; however, this also yields conflicting results [5,6,7,8,9,10,11].

There are several studies devoted to the search for markers for differentiating the disease based on non-invasive liquid biopsy [12,13,14,15], including mRNA profiling in urine. For example, HOXC6 and DLX1 mRNA levels in combination with patients’ clinical data and risk factors can better stratify patients by risk and identify patients with high-grade PCa [16]. In addition, it has been shown that analysis of the expression of a chimeric gene, *TMPRSS2*: *ERG*, in urine in combination with PCA3 level can predict both the presence of PCa and high-grade PCa on biopsy [17].

To improve the predictive value of tests in detecting disease aggressiveness, new biomarkers that can predict the course of the disease and be useful for making therapeutic decisions are needed. Small non-coding RNAs have the potential mentioned above. It is known that epigenetic mechanisms are activated in gene regulation at the earliest stages of carcinogenesis. The identification of those microRNAs (miRNAs) that can serve as predictors of PCa development and severity, aid in molecular classification, provide a prognosis, and be useful for personalizing patient management tactics while avoiding unnecessary treatment costs is a crucial task [18].

Data concerning miRNA expression analysis in PCa tissues are abundant, yet their results exhibit contradictions and limited reproducibility in other studies. Furthermore, the Volga-Ural region of the Eurasian continent remains an unexplored area in the miRNA expression profile of patients with PCa. To address this gap, we have undertaken a comprehensive miRNA expression screening study, utilizing microarrays on prostate adenocarcinoma tissues obtained during prostatectomy, as well as normal tissues. This study considers the disease stage and its progression degree, with the goal of identifying miRNAs with a crucial role in PCa pathogenesis and disease progression.

## 2. Materials and Methods

### 2.1. Tumor Samples and Data Collection

Tissue specimens from both cancerous and healthy prostate tissues were obtained from individuals diagnosed with prostate cancer who underwent treatment at the Republican Clinical Oncological Dispensary and the Departments of Oncology and Urology at the Clinic of Bashkir State Medical University over the period spanning from 2014 to 2020. An evaluation of these tissue samples was conducted by two proficient pathologists with expertise in prostate cancer. They examined the tissues to determine their histological cell type, TNM stage, and Fuhrman nuclear grade.

Written informed consent for the collection of biological liquids and tissues and participation in the scientific research was received from each patient. The study protocol received approval from the Research Ethics Committee of the Institute of Biochemistry and Genetics, a subdivision of the Ufa Federal Research Center of the Russian Academy of Sciences. The study was conducted in accordance with the ethical standards outlined in the Declaration of Helsinki by the World Association.

A high-throughput miRNA expression profiling using a TaqMan OpenArray Human MicroRNA panel was carried out on a group of 46 PCa patients. For the validation stage, we included 39 PCa patients who met the criteria for participation, which included a histologically confirmed diagnosis of PCa and a minimum of 80% tumor cells in each tumor sample. Clinical data, such as age, TNM stage, histological type, PSA level, Gleason Score, and Fuhrman grade, were collected. Additionally, these patients had not received chemotherapy or radiotherapy before the tissue sample collection. There were no restrictions based on age, ethnic origin, or stage of cancer for participation in the research.

### 2.2. miRNA Extraction, Reverse Transcription, Expression Analysis of 754 miRNAs and miRNAs Individual Assays

At the profiling stage, the reverse transcription of miRNAs was conducted to examine their varying expression levels. This process utilized the TaqMan^®®^ MicroRNA Reverse Transcription Kit, provided by Applied Biosystems (Waltham, MA, USA). The reaction mixture comprised 5 μL of total RNA, 7 μL of a master mixture containing reverse transcriptase, dNTPs, and other essential components, along with 3 μL of the 5X OT primer designed specifically for the targeted miRNA gene. The reverse transcription reaction was carried out according to the following conditions: 16 °C for 30 min, 42 °C for 30 min, 85 °C for 5 min, and finally held at 4 °C indefinitely. For real-time quantitative PCR analysis, the TaqMan MicroRNA Assays kit from Applied Biosystems and the CFX96™ Real-Time PCR Product Detection System from BioRad (Hercules, CA, USA) were used. All samples were analyzed in triplicate, and all experiments were performed at least three times. All experimental procedures and data analyses were performed following the methodology detailed in our previous publication [19].

### 2.3. Bioinformatic Analysis of the TCGA Data

With the view to identifying differentially expressed target genes of studied miRNAs, we performed a bioinformatics analysis of the data from TCGA for PCa (PRAD project). Patients of the European population were selected for analysis, and the analysis included two comparisons: 1—normal samples against localized PCa (N vs. LPCa; the earliest stage presented in the project), 2—normal samples against localized PCa + locally advanced PCa with the absence of lymphogenous metastasis (stage N0; N vs. LPCa + LAPCa). The inclusion of samples of locally advanced PCa with N1 will greatly confound gene expression results. The sample size comparison of N vs. LPCa amounted to 40 paired samples (normal/tumor), and the comparison of N vs. LPCa + LAPCa resulted in 74 paired samples (normal/tumor). In the analysis of the provided data, we conducted a differential gene expression assessment between two distinct groups, specifically the normal and tumor groups. This analysis was carried out using the edgeR package within the R program. Additionally, we utilized the multiMiR package to compile lists of target genes associated with selected microRNAs.

These lists of target genes were generated based on data sourced from miRecords, miRTarBase, and TarBase databases, all of which contain experimentally confirmed miRNA-target associations. Subsequently, we compared the lists of target genes for each microRNA with the outcomes of gene expression analysis, retaining only those genes for which statistical significance indicated a significant alteration in their expression levels within the tumor group (FDR tests > 0.05).

#### Statistical Analysis

Statistical analysis was performed using Expression Suite Software v.1.0.1 for the discovery phase. The data were normalized using global normalization, and gene expression was quantified using the 2^−ΔΔCt^ method. To normalize target miRNA expression, the means of three housekeeping miRNAs (RNU6, RNU44, and RNU48) were employed. For quantitative group comparisons, the equality of variance in the data distribution was assessed through the Mann–Whitney U-test. Statistical significance was determined using *p*-values, and we controlled for the false discovery rate (FDR) with the Benjamini–Hochberg method to address multiple testing. Calculations were carried out using GraphPad Prism 6.07 software and the R environment. To evaluate the discriminative abilities of logistic regression models, receiver operating characteristic (ROC) curves were employed in the R environment with the pROC package. The model’s capacity to distinguish between tumor tissue and normal prostate tissue was evaluated by comparing the area under the curve (AUC) of the respective ROC curves, which measures the true-positive rate versus the false-positive rate.

## 3. Results

**Figure 1:**
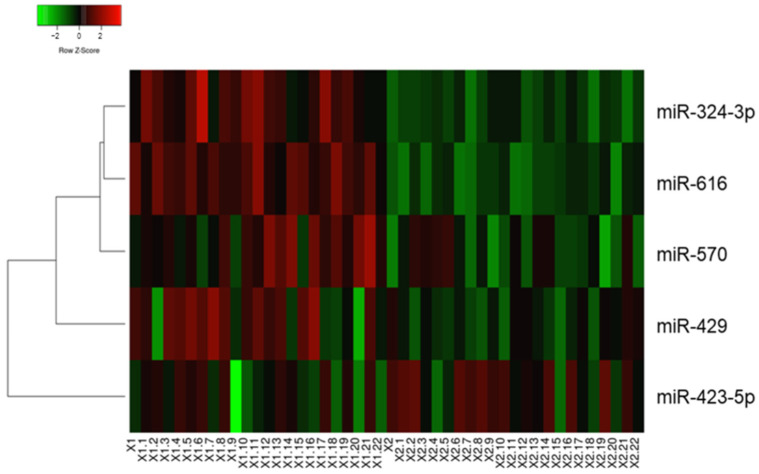
In the discovery phase, miRNAs with differential expression were identified in patients with prostate cancer. Group X1-X1.22 included late-stage prostate cancer tissues, while group X2-X2.22 included early-stage prostate cancer tissues.

The current study aimed to investigate miRNA profiles in patients with PCa (Table 1). In the discovery phase, 754 miRNAs were screened using microarrays in tissues obtained from patients with PCa in early (I–II TNM) and late (III–IV TNM) stages (23 samples in each group), and five differentially expressed miRNAs were identified. Expression levels of miRNA-324-3p, miRNA-429, miRNA-570, and miRNA-616 were found to be downregulated in patients with early-stage cancer compared to those with late-stage cancer. In contrast, miRNA-423-5p expression was upregulated in patients with early-stage cancer (Table 2). The heat map of the observed results is presented in Figure 1.

### 3.1. Validation Phase

**Figure 2:**
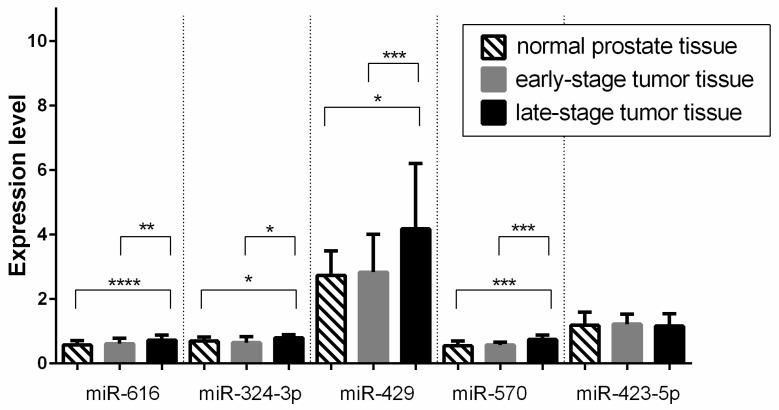
Analysis of miRNA expression in normal prostate tissue, as well as early and late stages of TNM in PCa tumor tissues, was performed on the validation group. The *p*-values were calculated using the Mann–Whitney test and the *t*-test, depending on the normality test results (*, *p* < 0.05, ** *p* < 0.01, *** *p* < 0.001, **** *p* < 0.0001). The bars are presented with a median and an interquartile range.

The five miRNAs found differentially expressed in the discovery phase were further validated in an independent cohort of samples (*n* = 39) collected from patients with PCa. MiRNA-324-3p, miRNA-429, miRNA-570, and miRNA-616 expression levels remained significantly downregulated in early-stage cancer tissues compared to late-stage tissues. However, miRNA-423-5p did not show a significant difference between the compared groups (Figure 2).

### 3.2. ROC Analysis

**Figure 3:**
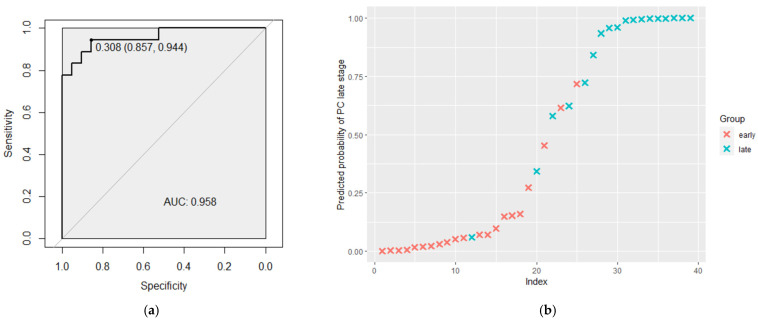
(**a**) The ROC curve regarding the significance expression level of three miRNAs, PSA levels and Gleason scores combination to discriminate PC stages. (**b**) The predicted probability of the model, to identify the stage of the tumor process along with the actual status. ROC—receiver operating characteristic; AUC—area under the curve; early—early stages of prostate cancer, late—late stages of prostate cancer.

An estimation of the distinguishing probability of four validated miRNAs and clinical predictors was performed using logistic regression analysis, following ROC analysis (Table 3). It was shown that the best model with the highest AUC (0.958) included three miRNAs, PSA levels, and Gleason scores and demonstrated sensitivity equal to 0.857 and specificity equal to 0.944. The positive predictive value, negative predictive value and number of correctly classified cases are presented in Table 4. In addition, the current model has a high possibility of predicting the stage of prostate tumors, as is shown in Figure 3. Models that include only miRNA combinations and PSA plus Gleason score combinations are presented in Supplementary File S8.

### 3.3. Identification of Prognosis-Related Variables

Multivariate Cox regression analysis was performed to screen out the most prominent variables, which could serve as a prognostic model (Table 5). It was observed that the most significant variables included the Gleason score, miR-616 and miR-324.

**Figure 4:**
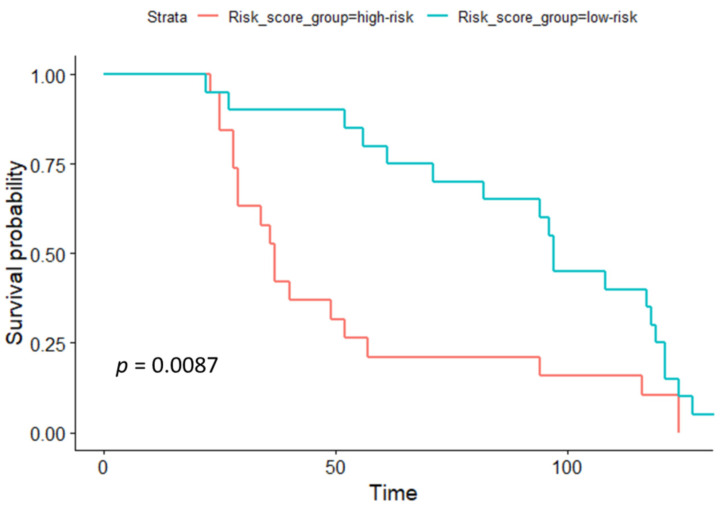
The prognostic model to predict the survival of the PCa. Kaplan–Meier survival curve unveils the survival rate of the high-risk group and low-risk group. “*p*” is the *p*-value of a log-rank test. High-risk and low-risk are groups of patients are divided according to the risk score value.

Next, the group of patients was divided into two subgroups, according to the risk score. Individual risk scores were calculated as follows: Risk score = 1.01 × PSA + 2.83 × Gleason score + 10.41 × miR-616 expression level + 12.43 × miR-324 expression level + 1.81 × miR-570 expression level. The median value was chosen as a cutoff for distinguishing high- and low-risk groups. Further, the survival curve of the risk score revealed that PCa patients with a high risk score had a poor prognosis (*p*  = 0.0087, Figure 4).

### 3.4. Pathway Enrichment Analysis

**Figure 5:**
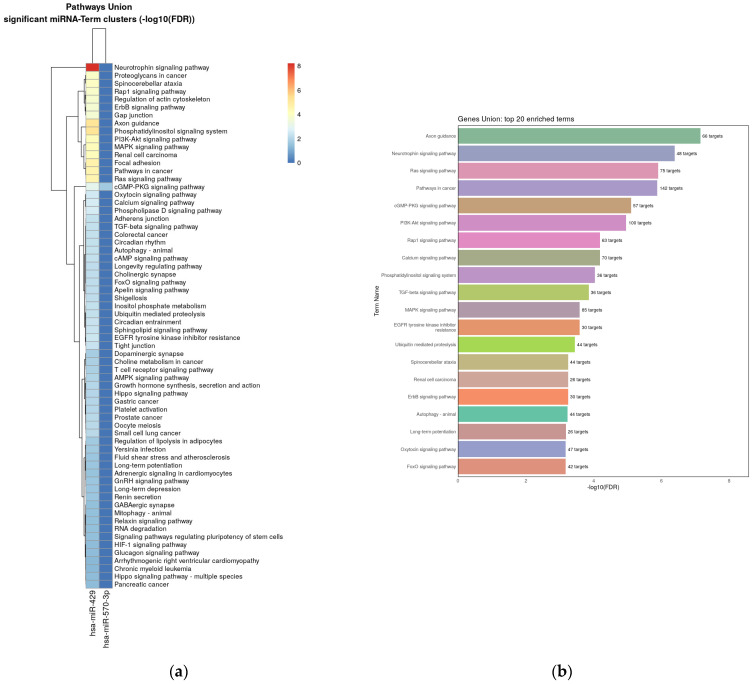
Heat map with targeted pathways clusters of KEEG pathways that include studied miRNAs target genes (based on microT-CDS algorithm): (**a**)—pathway union merging method; (**b**)—gene union merging method.

**Figure 6:**
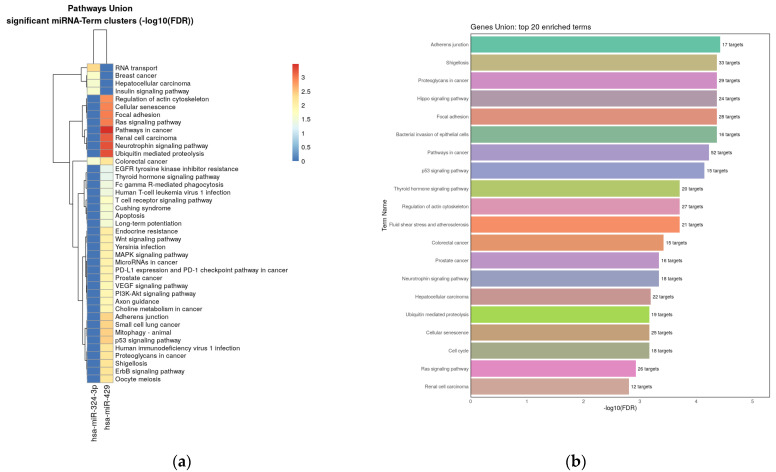
Heat map with targeted pathways clusters of KEEG pathways that include studied miRNAs target genes (based on Tarbase): (**a**)—pathway union merging method; (**b**)—gene union merging method.

Four validated miRNAs were analyzed in reference to their target genes using ShinyGo, String and miRPath v.4.0 from Diana Tools resources. MiRPath analysis using microT-CDS algorithm (the way to merge results is pathways union) demonstrated 67 KEEG pathways with potential targets of studied miRNAs for pathway union (Figure 5a) and 71 KEEG pathways for gene union (Figure 5b). Next miRPath analysis was performed based on Tarbase v8.0—a database of experimentally supported miRNA:gene interactions. It was shown that the studied miRNA targets involved 42 pathways for pathway union and 66 pathways for the gene union merging method, including the PI3K-Akt signaling pathway and the p53 signaling pathway, which are extremely important in cancer pathogenesis (Figure 6a,b). Interestingly, the most significant contribution to enrichment was provided by the target genes of miRNA-429 and miRNA-324-3p.

**Figure 7:**
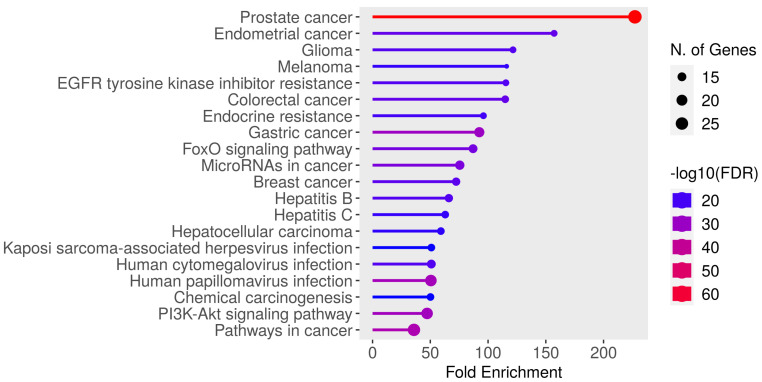
Top 20 pathways of prostate cancer pathway genes from ShinyGo enrichment analysis. N. of Genes—number of genes.

**Figure 8:**
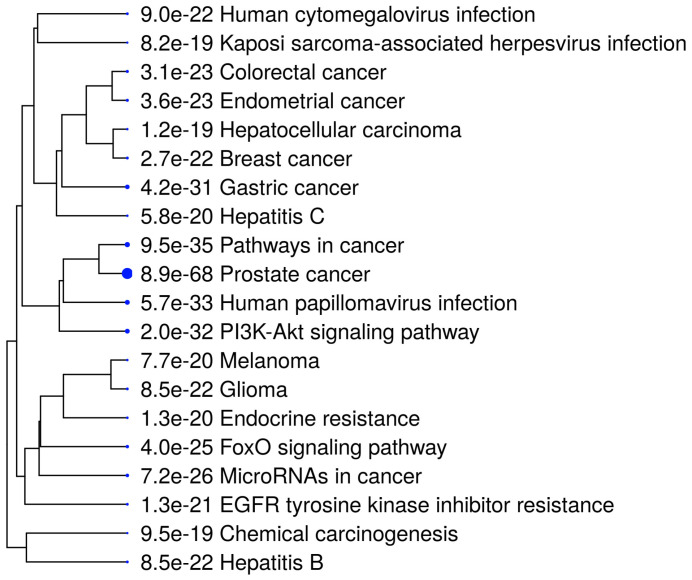
A hierarchical clustering tree summarizes the correlation among significant pathways of studied target genes. Pathways with many shared genes are clustered together. Bigger dots indicate more significant *p*-values.

**Figure 9:**
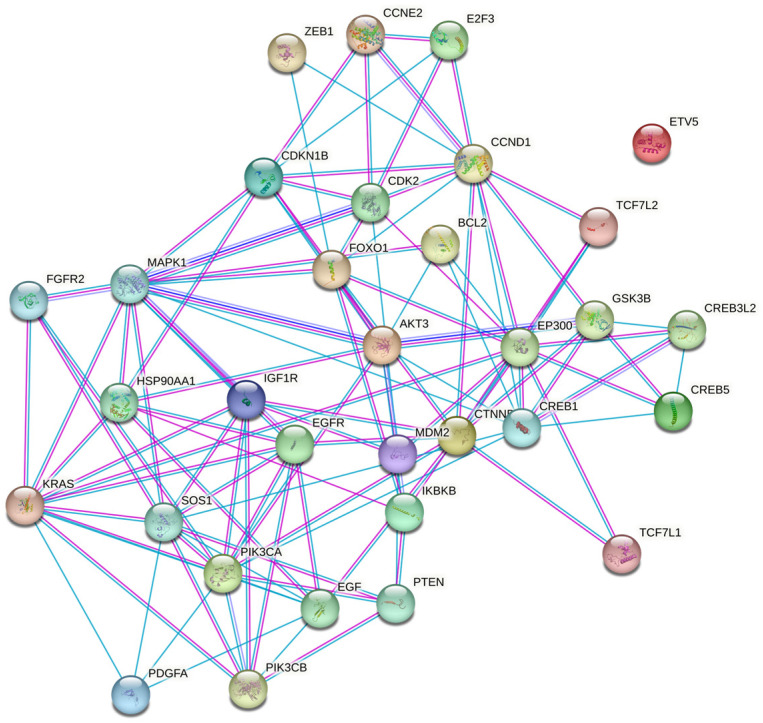
STRING nodes graph of eleven target genes of miRNA-429.

**Figure 10:**
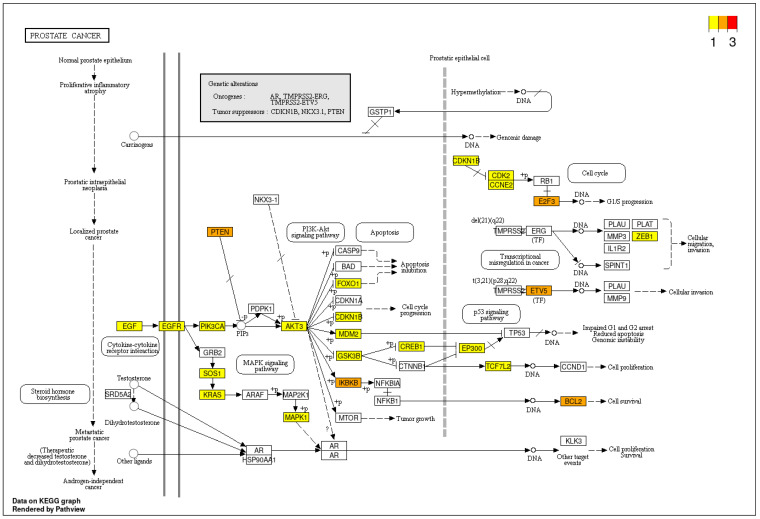
Schematic representation of prostate cancer molecular processes based on data from KEGG. Genes identified as miRNA-429 targets are highlighted in color: yellow—target of one miRNA, orange—target of two miRNAs, and red—target of three miRNAs.

For further analysis, we chose a set of 32 target genes from the prostate cancer pathway presented in each enrichment analysis (Tarbase and microT-CDS) (Figure 7, Supplementary File S1). As is seen in the hierarchical clustering tree of pathway correlation (Figure 8), the most significant clusters included the prostate cancer pathway, pathways in cancer, and the PI3K-Akt signaling pathway. STRING analysis PPI enrichment (*p*-value = 1.0 × 10^−16^) demonstrated that the members of this set of genes are participants of 150 pathways in particular combinations (Figure 9; Supplementary File S2). Thirty-one of them enter into a list of prostate cancer pathways with a *p*-value = 2.91 × 10^−66^ and a strength of enrichment of 2.3. The places of these genes in molecular processes of prostate cancer development are presented in Figure 10. In addition, according to WikiPathways, 13 genes are engaged in the miRNA regulation of prostate cancer signaling pathways (*p*-value 2.98 × 10^−6^; strength = 2.49) (Supplementary File S3).

### 3.5. Expression Analysis of miRNA Target Genes Based on TCGA Data

**Figure 11:**
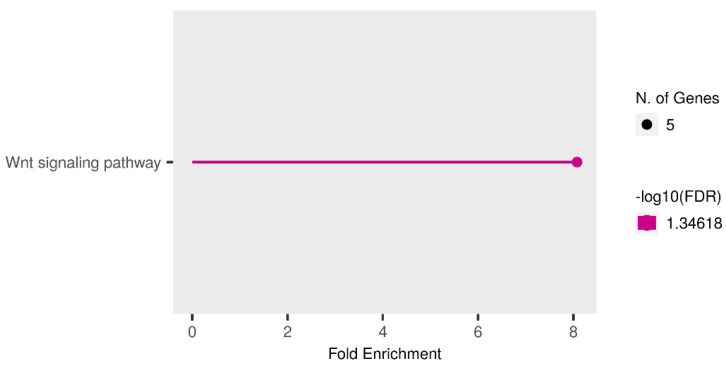
Enrichment pathways analysis for the downregulated target genes of miRNA-324-3p, miRNA-616, miRNA-570 and miR-429 based on a comparison of N vs. LPCa samples. Target genes with more than two-fold expression alteration were included in enrichment analysis using ShinyGo tools. N. of Genes—number of genes that participated in the pathway.

**Figure 12:**
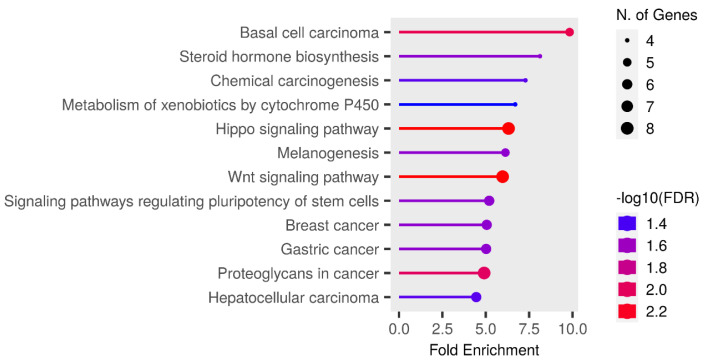
Enrichment pathways for the downregulated target genes of miRNA-324-3p, miRNA-616, miRNA-570 and miR-429 based on a comparison of N vs. LPCa + LAPCa samples. Target genes with more than two-fold expression alteration were included in enrichment analysis using ShinyGo tools. N. of Genes—number of genes that participated in the pathway.

Additionally, we performed a bioinformatic analysis of RNA-Seq data from TCGA for PCa (PRAD project). Patients of the European population were selected for analysis, and the sample size comparison of N vs. LPCa amounted to 40 paired samples (normal/tumor), while the sample size comparison of N vs. LPCa + LAPCa resulted in 74 paired samples (normal/tumor). The results are presented in the supplementary files results_Nvs.LPCa and results_Nvs.LPCa +LAPCa (Supplementary Files S6 and S7). Subsequently, target genes showing a negative correlation with miRNA expression were subjected to enrichment analysis using the ShinyGO online tool. The obtained enrichment analysis results are presented in Figure 11 and Figure 12 for the N vs. LPCa and N vs. LPCa + LAPCa groups, respectively. For the group of comparison between normal prostate tissues and localized PCa only, one pathway was enriched— the Wnt signaling pathway, which incsludes five target genes from the list of eighty-six target genes from TCGA. An enrichment analysis of comparison groups of normal tissues vs. localized PCa + locally advanced PCa with the absence of lymphogenous metastasis showed 12 significantly enriched pathways and 20 enriched pathways in total (Supplementary Files S4 and S5). As can be seen, most of the identified pathways refer to cancer development.

## 4. Discussion

In recent decades, the increasingly important roles of miRNAs in human diseases, especially cancers, have attracted more and more attention. For PCa, numerous miRNAs have been identified to regulate its tumorigenesis and metastasis, acting as oncogene or tumor suppressors. In a meta-analysis of 104 articles, encompassing multiple countries and predominantly Caucasian patients, various sample types (tissue, serum, plasma, urine) underwent miRNA expression analysis using qRT-PCR and microarrays [20]. This analysis identified 22 deregulated miRNAs in PCa, with miR-125b, miR-205, miR-1, and miR-23b showing the highest diagnostic efficiency. Elevated miR-10b, miR-100, miR-106b, miR-133b, miR-150, miR-191, miR-301a, miR-449b, miR-663, and miR-1207-3p correlated with reduced disease-free survival, while low levels of miR-23a/b, miR-27b, miR-34b, miR-224, miR-466, miR-709 and let-7b indicated poorer survival. Additionally, miR-32 and let-7c distinguished metastasis from primary PCa [20].

Another meta-analysis, involving 1371 participants across 10 studies in Poland, China, and Egypt, identified miR-21 as a robust diagnostic biomarker and miR-30c as a moderately effective diagnostic biomarker for PCa [21].

Previous studies have demonstrated that miR-324-3p participates in the regulation of tumor progression and EMT in various cancers, such as nasopharyngeal carcinoma, gastric cancer, hepatocellular carcinoma, and lung squamous cell carcinoma. However, the role of miR-324-3p in PCa cells remains unknown. For example, miR-324-3p was found to inhibit the proliferation and invasion of nasopharyngeal carcinoma cells through its negative regulation of the gene coding for zinc finger protein GLI3, with its expression being negatively related to the carcinogenesis, progression, and prognosis of the disease [22]. In this study, we found that miR-324-3p is downregulated in early-stage PCa compared to the late-stage tumors. It was previously shown that miRNA-324-3p could regulate the expression of oncogene *WNT2B.* It was suggested that WNT2B, as a member of the WNT2 protein family, plays a critical role in the canonical Wnt/β-catenin signaling pathway and affects various malignant tumor progressions. Moreover, the lncRNA SNHG7/miR-324-3p/WNT2B regulatory axis was supposed as a new therapeutic target for prostate cancer [23]. However, miR-324-3p was shown to be overexpressed in tumor tissues compared with adjacent normal tissues in gastric cancer cells, which was accompanied by promoted cell growth, migration, and decreased apoptosis. In that case, it was observed that miR-324-3p repressed the expression of Smad4, and the loss of Smad4 activated the Wnt/beta-catenin signaling pathway [24]. The tumor promotion of miR-324-3p through the Wnt/β-catenin pathway was also shown for hepatocellular carcinoma cells [25]. Contrariwise, in the experiments with breast cancer cells, it was shown that the overexpression of miR-324-3p inhibited cancer cell viability. This process was due to the interaction of miR-324-3p with *GPX4*, which led to the downregulation of *GPX4* and as a result, mediated the anti-cancer effect by promoting ferroptosis [26]. The same effect of miR-324-3p was observed for lung adenocarcinoma cells. First of all, it was found that miR-324-3p was significantly downregulated in the lung cancer cells. The *GPX4* gene was suggested as the direct target of miR-324-3p, and miR-324-3p expression restoration reversed the cancer cells’ sensitivity to cisplatin, again through inducing ferroptosis. As for plasma miR-324-3p, it was shown that this miRNA was upregulated in the early stages of lung squamous cell carcinoma, and in complex with miR-1285, was able to distinguish lung squamous cell carcinoma from lung adenocarcinoma, large-cell lung cancer and small-cell lung cancer [27].

The role of miR-616 in cancer development and progression is also controversial. Thus, it was shown that miR-616 promotes cancer progression, invasion, and migration in several cancers, such as bladder cancer, hepatocellular carcinoma, pancreatic carcinoma, and gastric and breast cancer [28,29,30,31,32]. One mechanism of miR-616 cancer promotion was suggested to be PI3K/AKT pathway activation through suppressing *PTEN* expression [30,31]. In prostate cancer, the oncogenic role of miR-616it was also demonstrated; specifically, it was shown that miR-616 is a target of circular RNA hsa_circ_0007494, which functioned as a “molecular sponge” for miR-616 and hence upregulates the target of miR-616, PTEN [33]. In addition, for miR-616, it was suggested that another circular RNA circ_0000673 could act as a sponge. It was shown that circ_0000673 knockdown could decrease the expression of *PTEN* and increase the expression of *PI3K* and *p-AKT* [34]. On the other hand, it was observed that in breast cancer, miR-616 could inhibit tumor cell growth and migration by suppressing *c-MYC* expression, demonstrating a tumor suppressor role [35].

Mir-570 also demonstrated ambiguous meaning for the oncogenesis. There is some evidence about the suppression role of miR-570, such as for colon cancer, where a low expression of miR-570 in colon cancer tissues was demonstrated, which indicated its correlation with poor overall survival, poor relapse-free survival, and unfavorable cancer-specific survival rates [36]. Likewise, the downregulation of miR-570 was shown in triple-negative breast cancer [37]. The tumor suppressive role of miR-570 was also demonstrated in hepatocellular cancer, where miR-570 mimics suppressed tumor growth and enhanced the ratio of CD8^+^IFN-γ^+^ T cells [38]. On the contrary, miR-570-3p was shown as a cancer-promoting gene in ovarian cancer. The activity of miR-570 again was shown to be regulated by circular RNA (circ_0007444), and PTEN was identified as a target gene of miR-570 [39]. In addition, it was mentioned that the miR-570 expression level has a negative correlation with *PD-L1* expression, which could be promising for the immunotherapy of cancers [39]. Recently, it was found that *PD-L1* is the target of miR-570, which contains binding sites in the 3′-untranslated regions (3′-UTR) of *PD-L1* [37].

Mir-429 is a widely studied miRNA, and many others have a dual role in cancer development. miR-429 is a member of the miR-200 family and is located on chromosome 1p36. Recently, it was shown that the downregulation of miR-429 could arrest the prostate cancer cell cycle in the G1 phase, which suggests the potential role of miR-429 in prostate cancer proliferation [40]. The upregulation of miR-429 was shown in endometrial carcinoma cells, and PTEN was suggested as a possible target of this miRNA [41]. In addition, exosomal miR-429 was demonstrated to enhance the proliferation and drug resistance of ovarian cancer cells by targeting the calcium-sensing receptor (CASR)/STAT3 pathway, which indicates that miR-429 may act as a primary regulator of chemoresistance [42]. In castration-resistant prostate cancer, serum miR-429 was shown to be upregulated and correlated with poor overall survival [43]. In clear cell renal cell carcinoma, it was found that a low expression of miR-429, negatively correlated with the overexpression of *CRKL*, (v-crk sarcoma virus CT10 oncogene homolog (avian)-like) promoted the aggressiveness of cancer cells and advanced the clinical progression of ccRCC patients. Additionally, miR-429 was suggested as a tumor suppressor in hepatocellular carcinoma by targeting *CRKL* via inhibiting the Raf/MEK/ERK pathway and the epithelial-mesenchymal transition, which leads to a decrease in tumor migration and invasion [44]. Also, as a tumor suppressor, miR-429 could inhibit the activation of the Wnt/β-catenin signaling pathway by targeting *HOXA9* in osteosarcoma [45] and *FN1* in breast cancer [46]. Moreover, the overexpression of miR-429, and other members of the miR-200 family (miR-200a/b/-429), produced a radio-sensitizing effect in tumor xenografts of prostate cancer, and miR-200a/b/-429 downregulation seems to predict cell adhesion-mediated tumor radio-resistance [47].

## 5. Conclusions

In summary, none of the markers alone has high specificity and sensitivity; therefore, test systems containing different markers are often used for the diagnosis and prognosis of diseases. Therefore, it is especially important to focus not only on PSA and the Gleason scale but also on other types of molecular markers that can support the molecular classification of PCa. According to the results we obtained in the current study, it could be suggested that miRNA-324-3p, miRNA-570 and miRNA-616, in combination with PSA and the Gleason scale, play a role in prostate cancer development and prognosis. Further extended studies are needed to establish a connection between the activity of these miRNAs and the mechanisms of prostate cancer pathogenesis.

## Figures and Tables

**Table 1:** Clinical and demographical characteristics of prostate cancer patients.

	Discovery Phase (*n* = 46)	Validation Phase (*n* = 39)
Age, mean (range)	65 (56–75)	63 (52–70)
Fuhrman grade		
2	20	19
3–4	26	20
TNM		
I–II	23	21
III–IV	23	18
Metastasis	10	8
PSA, mean (range)	16.02 (0.27–75.04)	14.3 (2.04–56.11)
Gleason Score		
<8	28	23
≥8	18	16

**Table 2:** Dysregulated miRNA expression levels from discovery phase.

miRNAs	Fold Change	P_FDR_-Value
miRNA-324-3p	0.67	<0.0001
miRNA-616	0.48	<0.0001
miRNA-570	0.82	0.0006
miRNA-429	0.39	0.0021
miRNA-423-5p	1.80	0.0145

**Table 3:** Results of logistic regression analysis for all studied predictors.

	Estimate	Std. Error	z Value	Pr (>|z|)
(Intercept)	−35.419466	12.487824	−2.836	0.00456
miR.616	0.009438	0.042614	0.221	0.82473
miR.324	3.047531	1.291730	2.359	0.01831
miR.570	5.923752	3.640018	1.627	0.10365
PSA	6.618535	4.263967	1.552	0.12061
Gleason_score	8.181656	3.991785	2.050	0.04040

**Table 4:** Predictive characteristics of miRNA, PSA and Gleason score-based model.

	Early Stage	Not Early (Late) Stage	Row Total	Predictive Values
Test Positive	18	1	19	PPV = 18/19 (94%)
Test Negative	3	17	20	NPV = 17/20 (85%)

PPV—positive predictive value; NPV—negative predictive value; early stage—early stages of prostate cancer, late stage—late stages of prostate cancer; Test positive—cumulative prediction score below cutoff; Test negative—cumulative prediction score above cutoff.

**Table 5:** The most striking variables identified via multivariate Cox regression analysis.

Variables	Coef	HR	HR.95 L	HR.95 H	*p*
PSA	0.01355	1.01364	0.9915	1.036	0.22904
Gleason_score	1.04361	2.83945	1.4593	5.525	0.00212
miR.616	2.34229	10.405	1.6563	65.365	0.01249
miR.324	2.52039	12.43345	1.6794	92.052	0.01361
miR.570	0.594	1.81122	0.3964	8.275	0.44349

## Data Availability

The data used and obtained during the study are available from the corresponding author on request.

## Supplementary Materials

The following supporting information can be downloaded at: https://www.mdpi.com/article/10.3390/biomedicines11113073/s1, Figure S1: (**a**) The ROC curve regarding the significance expression level of three miRNAs (miRNA-324-3p, miRNA-570 and miRNA-616) combination to discriminate PC stages. (**b**) The predicted probability of the model, to identify the stage of the tumor process along with the actual status. ROC—receiver operating characteristic; AUC—area under the curve; early—early stages of prostate cancer, late—late stages of prostate cancer. Figure S2: The predicted probability of the combination of PSA and Gleason score model, to identify the stage of the tumor process along with the actual status. early—early stages of prostate cancer, late—late stages of prostate cancer. File S1: Target genes from the prostate cancer pathway; File S2: Pathways from KEGG enrichment analysis; File S3: Pathways from WikiPathways enrichment analysis; File S4: The list of pathways from enrichment pathways analysis for the downregulated target genes of miRNA-324-3p, miRNA-616, miRNA-570 and miR-429 based on a comparison of N vs. LPCa samples; File S5: The list of pathways from enrichment pathways for the downregulated target genes of miRNA-324-3p, miRNA-616, miRNA-570 and miR-429 based on a comparison of N vs. LPCa + LAPCa samples; File S6: The results of bioinformatic analysis of RNA-Seq data from TCGA for PCa (PRAD project) based on a comparison of N vs. LPCa samples; File S7: The results of bioinformatic analysis of RNA-Seq data from TCGA for PCa (PRAD project) based on a comparison of N vs. LPCa + LAPCa samples; File S8: ROC analysis for the models based only miRNA combinations and PSA plus Gleason score combinations.
