# Supplementary material for: miRNA Expression Patterns in Early- and Late-Stage Prostate Cancer Patients: High-Throughput Analysis
Source: Biomedicines. 2023 Nov 16;11(11):3073. doi: 10.3390/biomedicines11113073 (PMC10669269; doi:10.3390/biomedicines11113073)
Supplement: Supplementary file 1 [file biomedicines-11-03073-s001.zip › File S8.pdf]

At table S1 are presented results of estimation of four validated miRNAs distinguishing probability using logistic regression analysis with following ROC analysis. In addition, current model has high possibility to predict the stage of prostate tumor as it shown at Figure S1. Positive predictive value, negative predictive value and number of correctly classified cases are presented in table S2.

Table S1 Results of logistic regression analysis for four validated miRNAs.

	Estimate	Std. Error	z value	Pr(>|z|)
(Intercept)	-10.720	3.382	-3.170	0.00152
miR-616	5.772	2.370	2.435	0.01490
miR-324-3p	5.470	2.768	1.976	0.04815
miR-570	4.200	2.445	1.718	0.08587

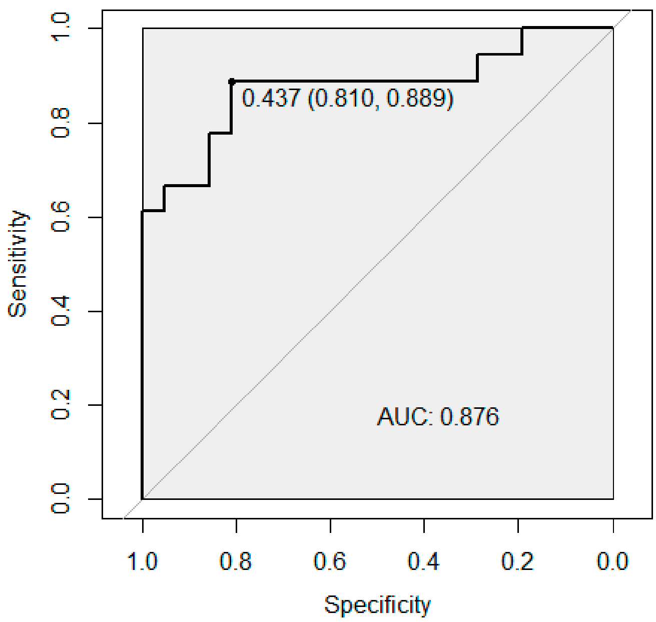

a)

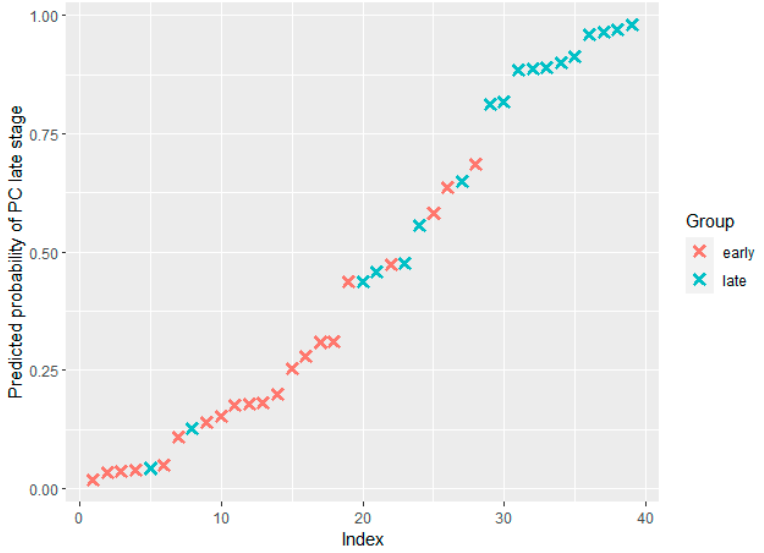

b)

Figure S1. (a)The ROC curve regarding the significance expression level of four validated miRNAs combination to discriminate PC stages. (b)The predicted probability of model to identify stage of tumor

process along with their actual status. ROC – receiver operating characteristic; AUC – area under the curve; early – early stages of prostate cancer, late – late stages of prostate cancer

Table S2. Predictive characteristics of four miRNA combination based model

	**Early stage**	**Not early (late) stage**	**Row Total**	**Predictive Values**
Test Positive	18	5	23	PPV = 18/23 (78%)
Test Negative	3	13	16	NPV = 13/16 (81%)

PPV- positive predictive value; NPV - negative predictive value; early stage - early stages of prostate cancer, late stage– late stages of prostate cancer; Test positive – cumulative prediction score down of cutoff; Test negative - cumulative prediction score above cutoff

At table S3 are presented results of estimation of PSA levels and Gleason scores combination distinguishing probability using logistic regression analysis with following ROC analysis. Current model has medium possibility to predict the stage of prostate tumor as it shown at Figure S2. Positive predictive value, negative predictive value and number of correctly classified cases are presented in table S4.

Table S2 Results of logistic regression analysis for four validated miRNAs.

	Estimate	Std. Error	z value	Pr(>|z|)
(Intercept)	-1.452e+01	4.808e+00	-3.020	0.00253
PSA	2.679e-04	2.583e-02	0.010	0.99173
Gleason_score	2.022e+00	6.911e-01	2.925	0.00344

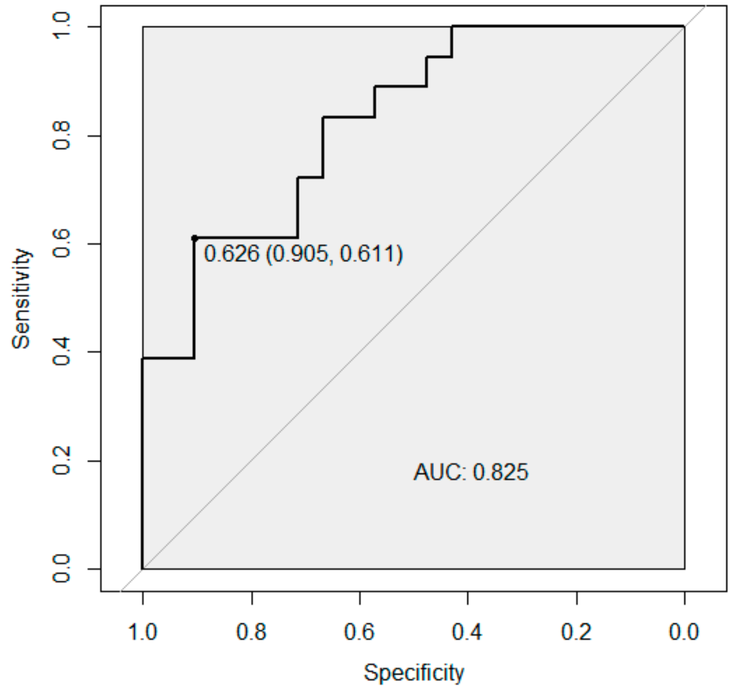

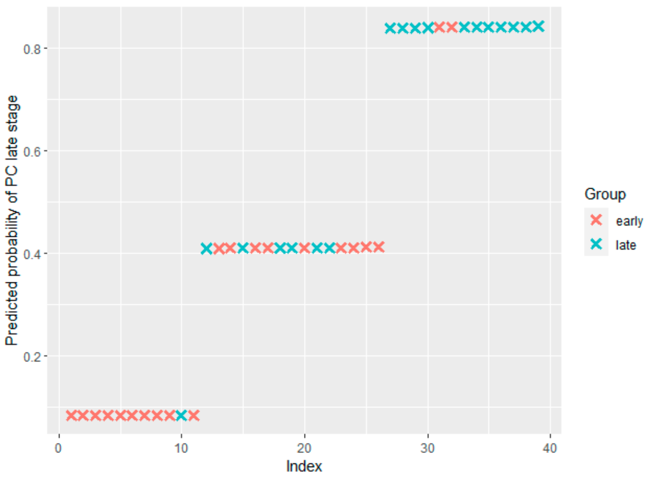

Figure S2. (a)The ROC curve regarding the significance expression level ofPSA and Gleason Score combination to discriminate PC stages. (b)The predicted probability of model to identify stage of tumor process along with their actual status. ROC – receiver operating characteristic; AUC – area under the curve; early – early stages of prostate cancer, late – late stages of prostate cancer

	**Early stage**	**Not early (late) stage**	**Row Total**	**Predictive Values**
Test Positive	19	7	26	PPV = 19/26 (73%)
Test Negative	2	11	13	NPV = 11/13 (85%)

PPV- positive predictive value; NPV - negative predictive value; early stage - early stages of prostate cancer, late stage– late stages of prostate cancer; Test positive – cumulative prediction score down of cutoff; Test negative - cumulative prediction score above cutoff
